# Multifocal Desmoplastic Small Round Cell Tumor: A Case Report of a Rare Neoplasm

**DOI:** 10.7759/cureus.53504

**Published:** 2024-02-03

**Authors:** Jyotirmoy Biswas, Rick Maity, Arkadeep Dhali, Sukanta Ray, Gopal Krishna Dhali

**Affiliations:** 1 General Medicine, College of Medicine & Sagore Dutta Hospital, Kolkata, IND; 2 Ophthalmology, Institute of Post Graduate Medical Education & Research, Kolkata, IND; 3 Gastrointestinal Surgery, School of Digestive and Liver Diseases, Institute of Post Graduate Medical Education & Research, Kolkata, IND; 4 Gastroenterology, School of Digestive and Liver Diseases, Institute of Post Graduate Medical Education & Research, Kolkata, IND

**Keywords:** metastasis, desmoplastic small round cell tumor, malignancy, orbit, dsrct

## Abstract

Desmoplastic small round cell tumor (DSRCT) is a rare, highly aggressive malignancy predominantly affecting adolescents and young adults. We report a case of multifocal DSRCT in an 11-year-old male who presented with complaints of unilateral forehead swelling, proptosis, and ophthalmoplegia for four months along with abdominal pain and dysphagia for six months. A whole-body computed tomography revealed widespread lesions in the skull, orbit, thorax, and abdomen with local infiltration. Ultrasound-guided biopsy of the forehead lump was performed. Based on histopathological and immunohistochemical investigations, it was diagnosed to be a DSRCT with multifocal presentation. The patient underwent chemo-radiation but unfortunately succumbed to neutropenic sepsis and renal failure. DSRCT is a very rare, highly aggressive malignancy with an extremely poor prognosis. Orbital presentations are even rarer, with less than 10 such cases currently described in English medical literature.

## Introduction

Desmoplastic small round cell tumor (DSRCT) was first identified by Gerald and Rosai in 1989. It is characterized by small round cells ensconced within an extensive desmoplastic stroma [1]. While the exact pathogenesis of DSRCT remains unknown, it is known to exhibit a unique translocation, t(11;22)(p13;q12), resulting in the formation of EWS-WT1 fusion protein, which is pathognomonic for this disease [2]. DSRCT typically arises in the abdominal and pelvic cavities and commonly presents with a combination of gastrointestinal symptoms such as abdominal pain, weight loss, and fatigue. We describe a case of multifocal DSRCT where the prominent complaint was unilateral forehead swelling, proptosis, and ophthalmoplegia along with abdominal pain and dysphagia. Given the scarcity of DSRCT cases, this report contributes to the existing knowledge base of atypical presentations of DSRCTs.

## Case presentation

**Figure 1:**
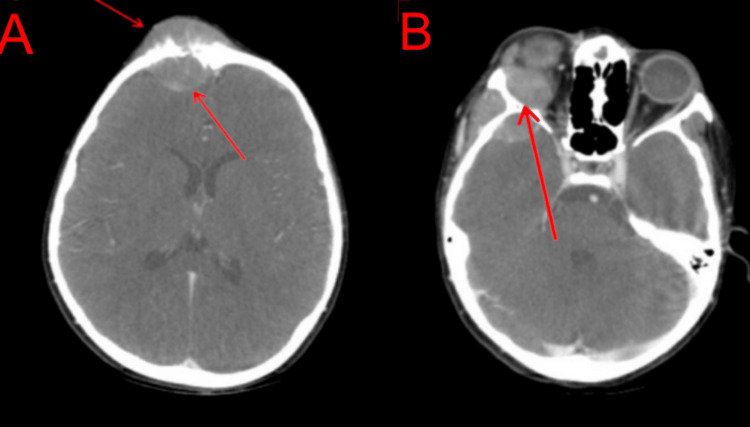
A: Contrast-enhanced CT showing enhancing soft tissue density mass involving the right half of the frontal bone with strong periosteal reaction and intracranial extension with effacement of the right frontal lobe. B: Lesion involving the right orbit and the right zygoma.

An 11-year-old boy with no significant family history presented with complaints of dull aching abdominal pain and difficulty swallowing solid food but denied any weight loss for six months. He also complained of swelling on the forehead and bulging of the right eye with a restriction of movements for the past four months. Physical examination revealed bronchial breath sounds over the left infra-clavicular and mammary areas with a vague mass felt in the umbilical and left lumbar area. Contrast-enhanced computed tomography of the head, neck, chest, and abdomen was done. It showed an enhancing soft tissue density mass involving the right half of the frontal bone with a strong periosteal reaction and intracranial extension with effacement of the right frontal lobe (Figure 1A). The soft tissue component also involved the right orbit and the right zygoma (Figure 1B).

**Figure 2:**
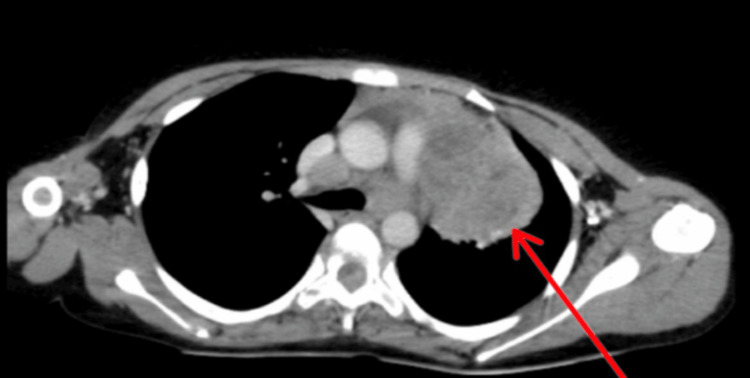
Contrast-enhanced CT showing mediastinal lesion with encasement of the arch of the aorta and bilateral pulmonary arteries.

**Figure 3:**
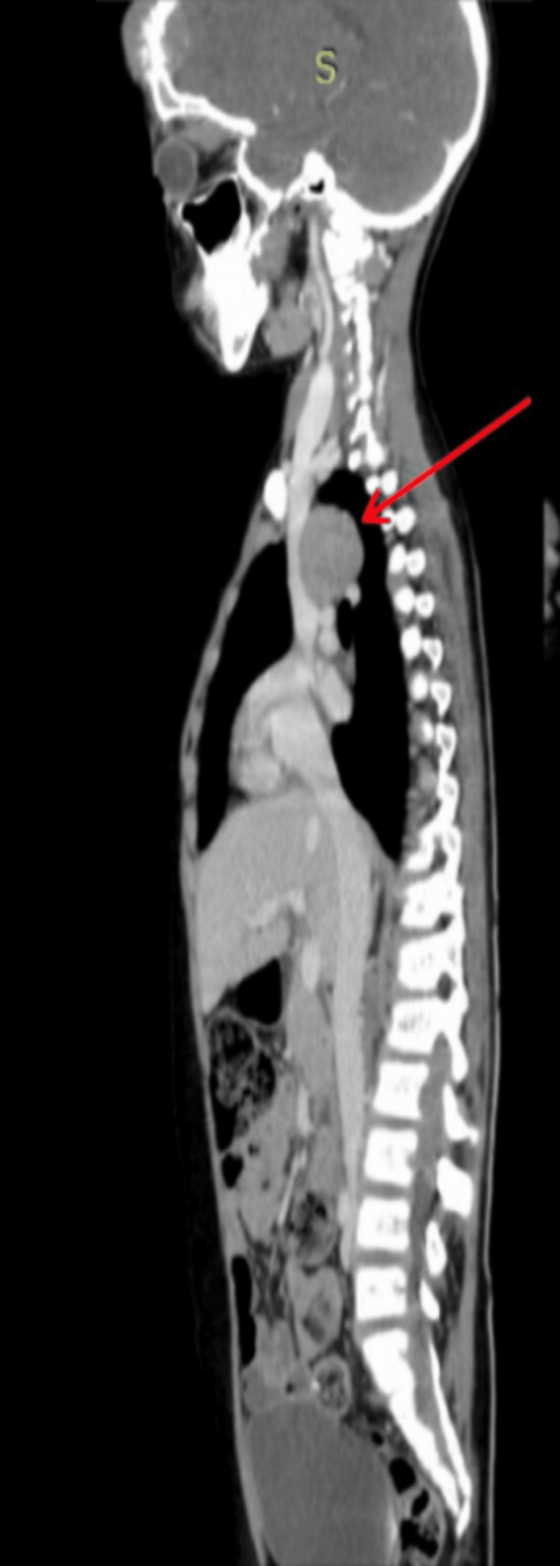
Contrast-enhanced CT showing lesion compressing the esophagus posteriorly.

In the thorax, a large, enhancing, soft tissue mass was seen in the paraesophageal area with encasement of the arch of the aorta and bilateral pulmonary arteries (Figure 2) and compressing the esophagus posteriorly (Figure 3).

**Figure 4:**
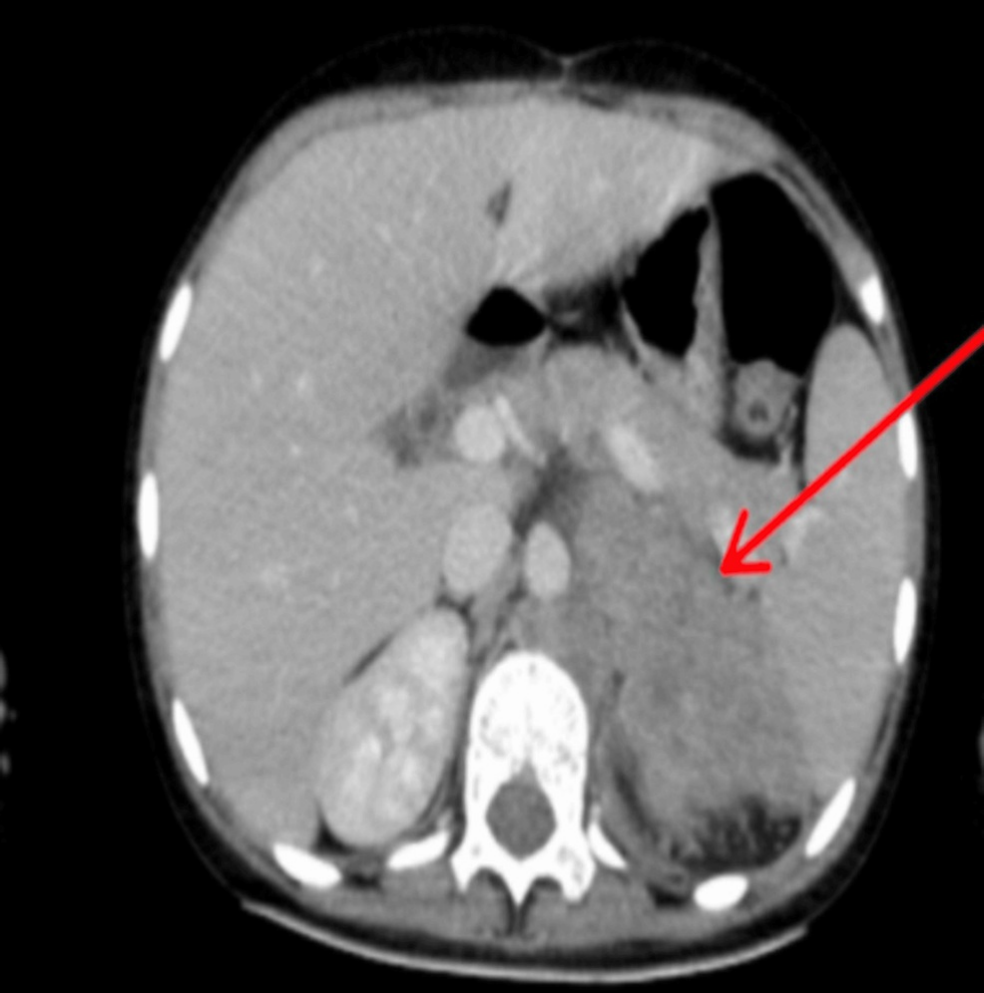
Contrast-enhanced CT showing an enhancing mass of the left adrenal gland with retroperitoneal enhancing lymph nodes noted with a partial aortic abutment.

In the abdomen, an enhancing mass of the left adrenal gland with retroperitoneal enhancing lymph nodes was noted with a partial aortic abutment (Figure 4).

**Figure 5:**
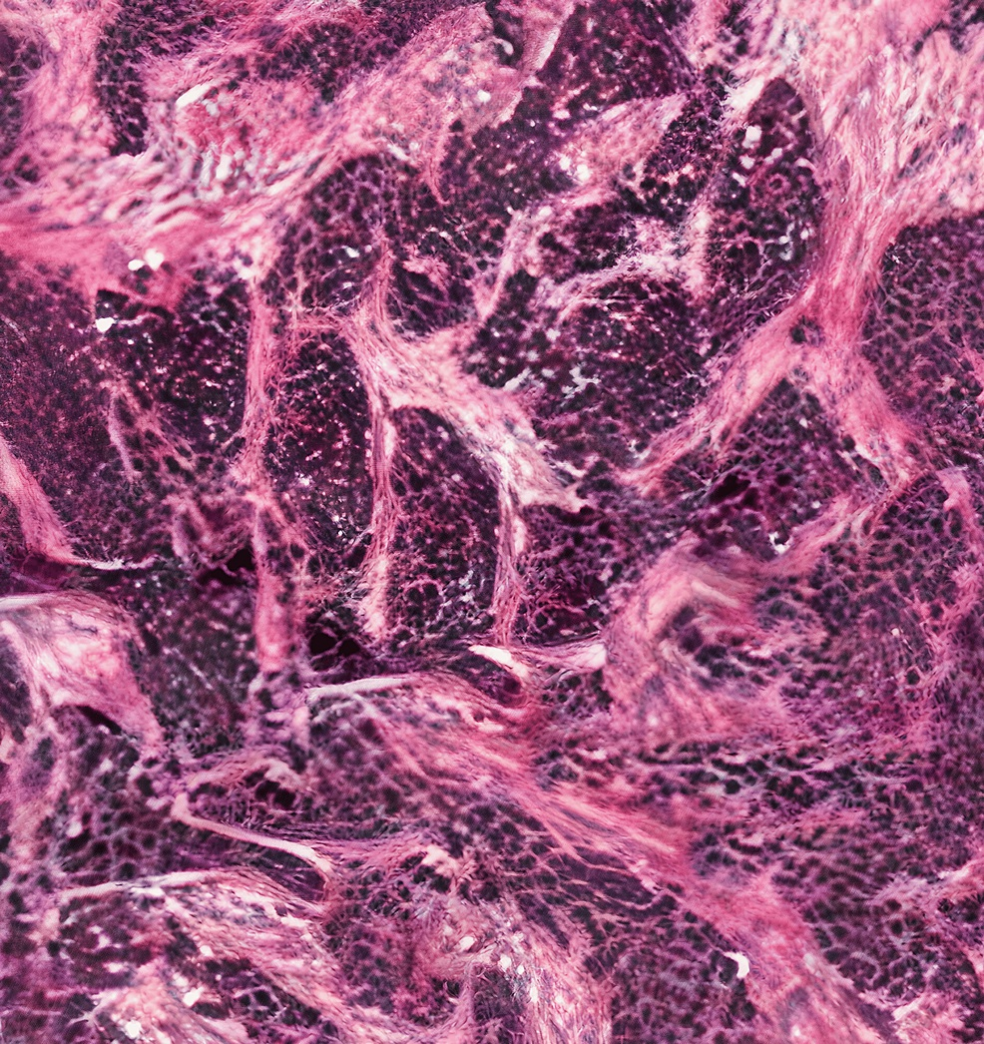
Hematoxylin & eosin image (200x) showing cohesive nests of undifferentiated small blue cells within the fibrocollagenous stroma, suggestive of desmoplastic small round cell tumor.

Given the age, initial presentation, and imaging findings, it raised suspicions of an aggressive disease, possibly a metastatic disease, midline tumors, or tumor syndromes, leading to the consideration of multiple differentials. These differentials included but were not limited to aggressive squamous cell carcinoma, NUT midline carcinoma, Cowden syndrome, neuroendocrine tumors, and carcinoma of unknown primary. The histopathological evaluation of the lump on the forehead by an ultrasound-guided biopsy revealed cohesive nests of undifferentiated small blue cells within the fibrocollagenous stroma (Figure 5).

Further confirmation was obtained through immunohistochemistry, which demonstrated positivity for cytokeratin and epithelial membrane antigen (EMA), suggesting DSRCT. He received one cycle of chemo-radiation but unfortunately succumbed due to neutropenic sepsis and renal failure within two months of the diagnosis.

## Discussion

In 1989, Gerald et al. discovered a malignant intra-abdominal tumor in an eight-year-old girl presenting with abdominal distension [1]. It was formally named desmoplastic small round cell tumor in 1991 since it was histologically composed of clusters of undifferentiated small round cells surrounded by abundant desmoplasia [3]. It is a rare and highly aggressive mesenchymal malignancy primarily affecting adolescents and young adults and predominantly affecting male patients [2]. DSRCT is thought to arise from the serosal surface of the abdominal cavity like the peritoneum. The signs and symptoms are non-specific and depend on the location of the tumor. The common presentations of this disease include the following in decreasing order of frequency: abdominal pain, abdominal discomfort, constipation, abdominal mass, and back pain. However, many patients develop widespread metastases at the time of diagnosis both inside and outside the abdominal cavity such as the liver, pancreas, kidneys, lungs, and rarely the cranium [4].

In our case, the main complaints were right-sided forehead swelling, unilateral proptosis, and ophthalmoplegia, along with dull aching abdominal pain and dysphagia to solid food, which were quite diverse and hard to point at a unifying diagnosis.

DSRCTs in the head and neck are very uncommon, orbital involvements being extremely rare. Less than 10 such cases are discussed in the current literature [5-8]. Due to the highly aggressive nature of the disease and the short duration of history, it is difficult to locate the primary site of origin of the tumor [2].

Hayes-Jordan et al. proposed a staging system for DSRCT where stage 1 would have patients with localized disease, limited to one or two abdominal sites; stage 2 would include extensive peritoneal disease; stage 3 with peritoneal disease and liver metastasis; and stage 4 with disease that has spread outside the abdominal cavity, including the lymph nodes [9].

While the exact pathogenesis of DSRCT has not been established, chromosomal translocation t(11; 22)(p13; q12) leading to the fusion of Ewing sarcoma RNA-binding protein 1 (EWSR1) and Wilms tumor suppressor (WT1) genes is the hallmark event that causes upregulation of various growth factor genes (especially, PDGFRα and vascular endothelial growth factor) and transcriptional factors related to tumorigenesis [10,11]. This translocation is pathognomonic of DSRCT and can differentiate it from other small round cell neoplasms, such as Ewing’s sarcoma, small cell carcinoma, and mesothelioma [12]. Histologically, DSRCTs are typically characterized by cohesive nests of blue-staining small, round, ovoid cells in a dense desmoplastic, collagenous stroma [13]. It is important to note that the tumor cells are positive for mesenchymal markers (desmin, vimentin), epithelial markers (cytokeratin, EMA), and neural markers (neuron-specific enolase, CD57), thereby displaying a unique triphenotype immunohistochemical profile [3,13,14].

Currently, there is no standard first-line treatment for DSRCT. In patients without extra-peritoneal metastases, a multimodal approach consisting of systemic chemotherapy, aggressive surgery, and adjuvant radiotherapy may prolong their survival [15,16]. As the disease is frequently diagnosed at an advanced stage, the Chicago consensus on peritoneal surface malignancies recommends starting treatment with chemotherapy since the tumor is chemosensitive [16]. Other treatment modalities such as hyperthermic intraperitoneal chemotherapy (HIPEC) post cytoreductive surgery and radioimmunotherapy (monoclonal antibody 131I-omburtamab targeting antigen B7H3 expressed on DSRCT tumor cells) are being explored, but no consensus has been reached regarding their standardized application in DSRCT [17-19]. Despite an aggressive multimodal therapeutic approach, the prognosis and survival rate remain poor, with overall survival ranging between 17 and 60 months and a five-year overall survival rate of 10-20% [20]. It is important to develop early detection strategies and establish standardized treatment guidelines to improve the outcomes.

## Conclusions

DSRCT is a very rare, highly aggressive malignancy with an extremely poor prognosis. Orbital presentations are even rarer, with less than 10 such cases currently described in the English medical literature. Despite no consensus on first-line management, a combination of chemotherapy, surgery, and radiotherapy has been proven to provide some survival benefits to the patient. Further longitudinal studies are required to develop strategies for early diagnoses and management plans based on the spread of the disease.
